# The evaluation of the efficacy and safety about apatinib combined with immune checkpoint inhibitors in advanced gastric cancer: a real-world study

**DOI:** 10.3389/fonc.2025.1578011

**Published:** 2025-06-06

**Authors:** Peng-Fei Zhu, Liu Yang, Zhe-Ling Chen

**Affiliations:** Cancer Center, Department of Medical Oncology, Zhejiang Provincial People’s Hospital, Affiliated People’s Hospital, Hangzhou Medical College, Hangzhou, Zhejiang, China

**Keywords:** gastric cancer, immunotherapy, apatinib, survival time, prognosis, antiangiogenesis

## Abstract

**Background:**

Apatinib and immune checkpoint inhibitors (ICIs) have shown promise as third-line treatments for advanced gastric cancer (AGC). This study compared the efficacy and safety of apatinib combined with ICIs versus apatinib monotherapy in AGC patients after second-line treatment failure.

**Methods:**

We conducted a retrospective analysis of 48 AGC patients with postoperative recurrence/metastasis treated at Zhejiang Provincial People’s Hospital between January 2018 and September 2022. Patients received either apatinib plus ICIs (n=23) or apatinib alone (n=25). Primary endpoints were overall survival (OS) and progression-free survival (PFS); Secondary endpoints included safety and subgroup analyses.

**Results:**

With median follow-up of 4.25 months, the combination group showed significantly longer median OS (6.0 vs 3.0 months, HR=0.44, 95%CI 0.24-0.82, *P*=0.009) and PFS (3.0 vs 2.0 months, *P*=0.155). Subgroup analysis revealed patients with liver metastasis receiving combination therapy had superior OS (7.5 vs 4.0 months, *P*=0.036). The objective response rate was higher with combination therapy (4.3% vs 0%), though not statistically significant (*P*=0.292). Safety profiles were comparable between groups, with no significant increase in severe adverse events with combination therapy.

**Conclusion:**

Apatinib combined with ICIs demonstrated improved survival outcomes compared to apatinib monotherapy in AGC, particularly for patients with liver metastasis, without increasing severe toxicity.

## Introduction

GC is prevalent malignant tumor in the digestive system. According to global cancer data from 2020, there were over 1 million new cases and approximately 769,000 deaths, ranking GC fifth in incidence and fourth in mortality worldwide (1). Apatinib and PD-1 monoclonal antibodies (mAb) have been confirmed to offer significant clinical benefits for AGC patients (2–5). Apatinib, a small-molecule tyrosine kinase inhibitor, primarily targets VEGFR2 but exhibits even stronger inhibition of c-Kit, potentially enhancing its anti-tumor effects through dual angiogenic and stromal modulation (6, 7). In 2017, the Chinese Society of Clinical Oncology (CSCO) guidelines for GC included apatinib as a recommended third-line therapy. Current evidence indicates that combining antiangiogenic agents with ICIs can significantly improve OS in patients with advanced solid tumors (8). Recent animal studies utilizing single-cell analysis have shown that apatinib can reshape the tumor microenvironment (TME) and enhance the efficacy of PD-1 mAb therapy (9). In a Phase I trial, the combination of the PD-1 mAb (SHR-1210) and apatinib in patients with advanced hepatocellular carcinoma (HCC), GC, or esophagogastric junction cancer (EGJC) demonstrated a median PFS of 2.9 months and a median OS of 11.4 months (10). Furthermore, a Phase II study demonstrated that second-line therapy with the PD-1 mAb (SHR-1210) combined with apatinib and S-1 in AGC yielded an objective response rate (ORR) of 29.2%, a mPFS of 6.5 months, and a manageable safety profile (11). The CSCO guidelines recommend a first-line treatment regimen for AGC consisting of fluorouracil-based chemotherapy combined with platinum-based agents. For HER2-positive patients, trastuzumab is incorporated into the regimen. Among HER2-negative patients, those with a PD-L1 combined positive score (CPS) ≥5 may receive nivolumab, while those with PD-L1 CPS ≥1 may be considered for pembrolizumab. Patients with microsatellite instability-high (MSI-H) or deficient mismatch repair (dMMR) tumors are eligible for immunotherapy regardless of PD-L1 status. For second-line therapy, the anti-angiogenic agents ramucirumab or fruquintinib may be administered in combination with paclitaxel. In third-line or subsequent treatment settings, both apatinib and PD-1 monoclonal antibodies are recommended therapeutic options. Currently, no standardized treatment regimen exists for AGC beyond third-line therapy. This study aims to evaluate the efficacy and safety of apatinib combined with ICIs in the third-line treatment of AGC.

## Methods

### Patient selection and study design

**Figure 1:**
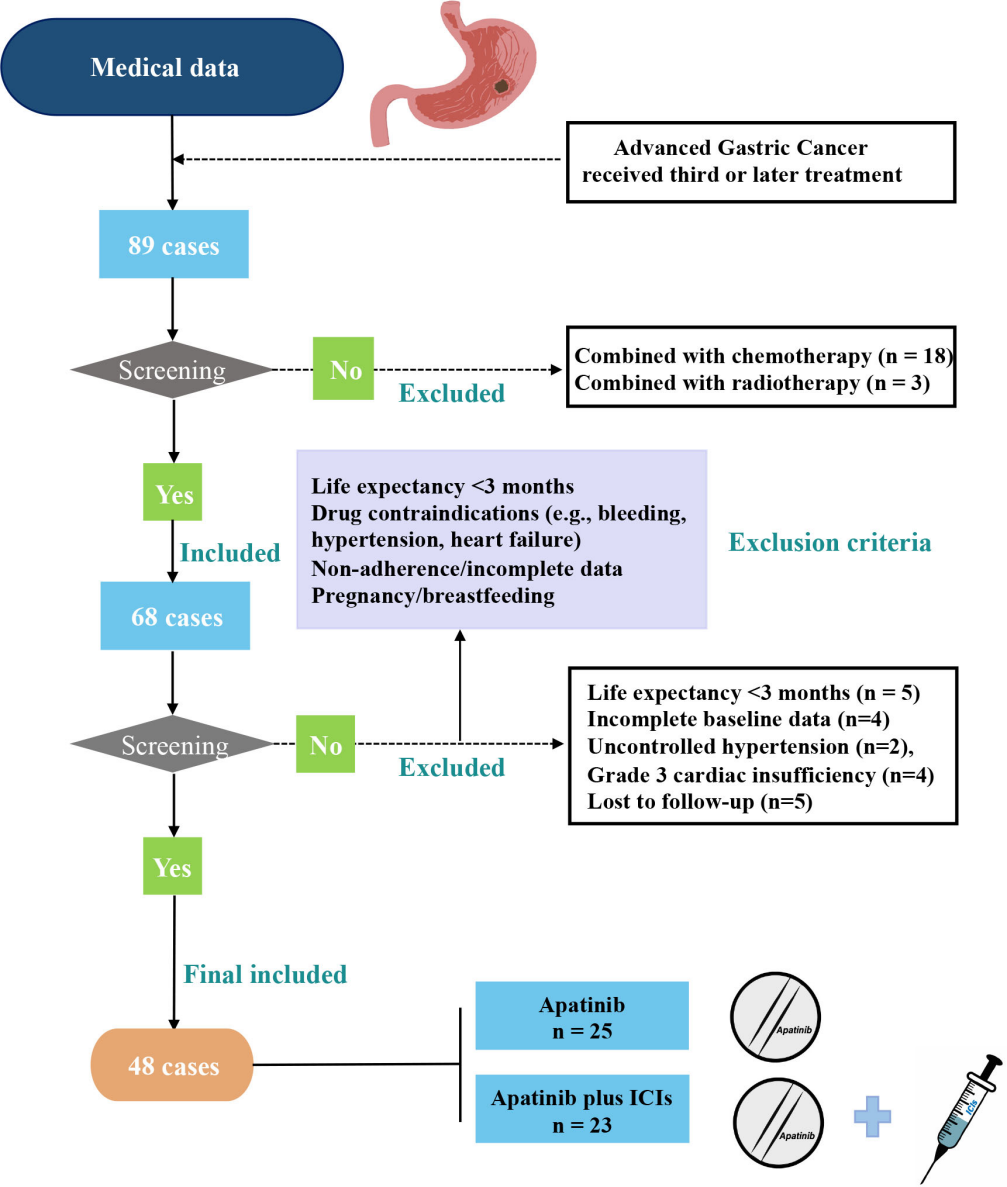
Patient inclusion flowchart.

This retrospective study evaluated 48 AGC patients treated with third-line therapy at Zhejiang Provincial People’s Hospital (July 2018-June 2022), including 23 receiving apatinib plus ICIs and 25 receiving apatinib monotherapy (**Figure 1**). Eligible patients met the following criteria: (1) histologically confirmed AGC; (2) age 18–75 years; (3) available efficacy/follow-up data; (4) failed/intolerant to second-line chemotherapy. Exclusion criteria were: (1) life expectancy <3 months; (2) treatment contraindications (active gastrointestinal bleeding, uncontrolled hypertension, or NYHA class III-IV heart failure); (3) incomplete records/lost to follow-up; (4) pregnancy/lactation. Ethical approval for this study was granted by the Ethics Committee of Zhejiang Provincial People’s Hospital. Our study was conducted in accordance with the guidelines outlined in the Declaration of Helsinki. As this was a retrospective study, the requirement for informed consent was waived.

### Treatment

In this study, a total of 48 patients were divided into two groups based on the use of ICIs: the apatinib monotherapy group and the apatinib combined with ICIs group. This treatment was primarily administered to patients with AGC who experienced disease progression or relapse following standard treatment, with a treatment cycle of 21 days. The standard dose of apatinib is 500 mg once daily. In our study, we tailored the dosing regimen based on patient-specific factors:500 mg/day was initiated for younger patients (age ≤65 years) with good performance status (ECOG 0-1), adequate organ function, and no uncontrolled comorbidities (e.g., hypertension <140/90 mmHg, proteinuria <1+);250 mg/day was used for patients with any of the following: ECOG ≥2, pre-existing grade ≥2 hypertension, cardiac dysfunction (LVEF <50%), hepatic impairment (Child-Pugh B), or prior intolerance to anti-angiogenic therapies. Dose adjustments were permitted based on toxicity management per protocol. All immunotherapy agents were PD-1 antibodies, including camrelizumab (n=14, 60.9%), nivolumab (n=6, 26.1%), and pembrolizumab (n=3, 13.0%), administered at standard doses (200 mg IV q2w or 240 mg IV q2w).

### Survival data collection

Survival data were obtained from: (1) hospital records, (2) quarterly telephone follow-ups, and (3) death registry verification. Two blinded investigators independently confirmed all endpoints, resolving discrepancies through physician consultation. Lost-to-follow-up cases (after ≥3 contact attempts) were censored at last verified contact.

### Statistical analysis

R version (4.2.0) was used for data processing and analyses. As appropriate, clinical characteristics between the two groups were examined via the t test, Wilcoxon rank-sum test, or *χ2* test. The treatment response was compared between the two groups by using the *χ2* test. The survival curves, including PFS and OS, were constructed by using the Kaplan–Meier method, and the variance analysis was determined via the log-rank test. ORR was defined as the rate of CR and PR among all the patients included. DCR was defined as the rate of CR and PR and SD among all the patients included. The prognostic analysis was completed by using univariable and multivariable Cox proportional hazard regression methods. Patients with metastases to multiple sites (e.g., liver and peritoneum) were included in all relevant subgroup analyses. For example, a patient with both liver and peritoneal metastases contributed to both the liver metastasis subgroup and peritoneal metastasis subgroup. *P*-value < 0.05 indicated a statistically significant difference.

## Results

### Clinical characteristics

The clinical characteristics of combined treatment group and monotherapy group in the **Table 1** included age (≤45/45-60/≥60), gender (Male/Female), primary tumor site (Cardiac/Gastric body/others), ECOG score (0-1/≥2), liver metastasis (Yes/No), peritoneal metastasis (Yes/No), combined signet-ring cell carcinoma (Yes/No), initial dosage of apatinib (250mg/500mg).

**Table 1:** Patients baseline characteristics.

Factors	Apatinib	Apatinib Plus ICIs	*P*
	(N = 25)	(N = 23)	
Age			0.138
≤45	2	3	
45-60	11	4	
≥60	12	16	
Gender			0.753
Female	12	10	
Male	13	13	
Primary Tumor Site			0.197
Cardiac	6	4	
Gastric body	9	4	
others	10	15	
ECOG Score			0.018
0-1	10	17	
2	15	6	
Liver metastasis			0.263
Yes	8	11	
No	17	12	
Peritoneal metastasis			0.990
Yes	12	11	
No	13	12	
Signet-ring cell carcinoma			0.897
Yes	4	4	
No	21	19	
Adjuvant Chemotherapy			
Fluorouracil-only	6	5	
Platinum-only	0	0	
Fluorouracil+Platinum	12	10	0.820
None	7	8	
Prior 1st-line for recurrence			
Platinum-based	25	23	1.000
Prior 2nd-line for recurrence			
**Taxane-based regimens**	20	18	0.870
Paclitaxel ± Ramucirumab	18	16	
Docetaxel ± Ramucirumab	2	2	
**Anti-angiogenic monotherapy**	5	5	
Ramucirumab alone	5	5	
Initial dosage (Apatinib)			
250mg	13	21	0.002
500mg	12	2	

“Others” included: gastric antrum, gastroesophageal junction/fundus, diffuse-type.

Dose selection criteria: 500 mg for ECOG 0-1 + no comorbidities; 250 mg for ECOG ≥2 and/or significant comorbidities (hypertension, hepatic/renal dysfunction).

### Comparison of treatment response

The combination therapy group (n=23) demonstrated response rates of 0% CR, 4.3% PR (1 patient), 73.9% SD (16 patients), and 21.7% PD (5 patients), while the monotherapy group (n=25) showed 0% CR, 0% PR, 60% SD (15 patients), and 40% PD (10 patients). Although the combination group exhibited numerically higher objective response rates (ORR 4.3% vs 0%, *P*=0.292) and disease control rates (DCR 78.3% vs 60%, *P*=0.172) compared to monotherapy, these differences did not achieve statistical significance. The results suggest a potential clinical benefit of combination therapy, though further investigation is warranted to confirm these observations (**Table 2**).

**Table 2:** Comparison of therapeutic effects between the two groups.

Treatment Response (%)	Apatinib(N=25)	Apatinib + ICIs(N=23)	*P*
CR	0	0	
PR	0	1(4.3%)	
SD	15(60%)	17(73.9%)	
PD	10(40%)	5(21.7%)	
ORR	0	1(4.3%)	0.292
DCR	15(60%)	18(78.3%)	0.172

### Comparison of PFS and OS

**Figure 2:**
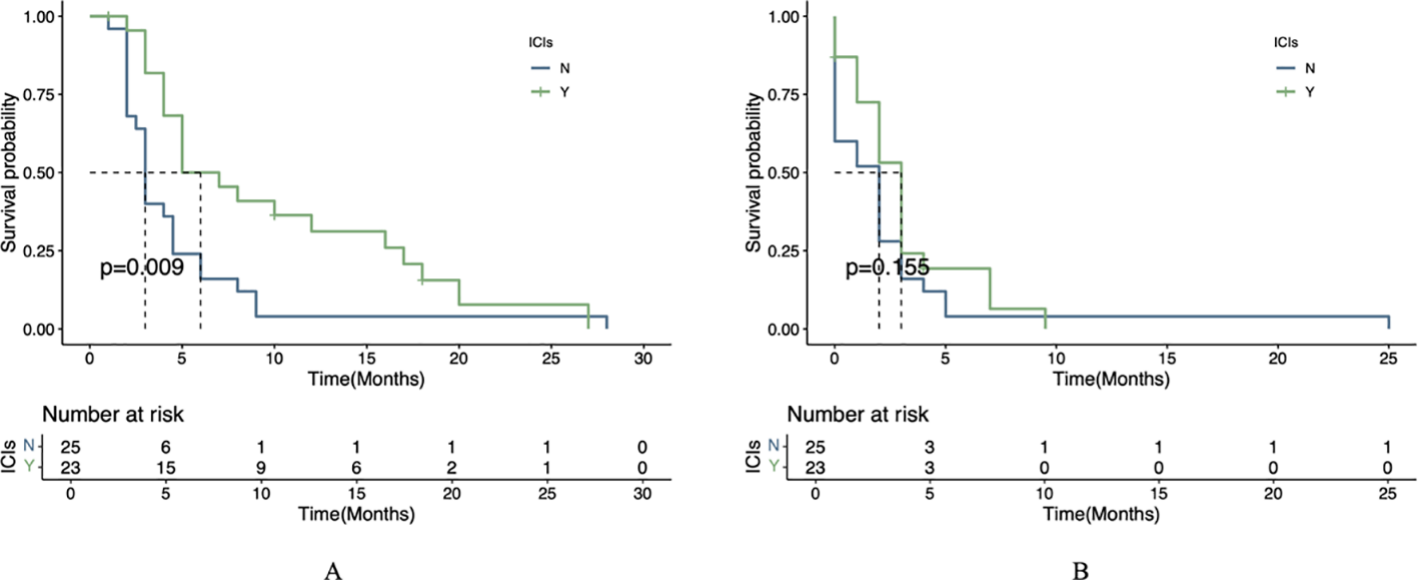
Survival outcomes by treatment regimen. **(A)** Kaplan-Meier curve of OS between the two treatment groups. **(B)** Kaplan-Meier curve of PFS between the two treatment groups.

The mOS was significantly longer in the combination therapy group (6 months, 95% CI 5.0-17.0) compared to the monotherapy group (3 months, 95% CI 2.5-4.5), with this difference reaching statistical significance (*P*=0.009; **Figure 2A**). For PFS, the combination group showed a numerically longer median PFS (3 months, 95% CI 2.0-4.0) versus the monotherapy group (2 months, 95% CI 0-3), though this difference did not achieve statistical significance (*P*=0.155; **Figure 2B**).

### Analysis of treatment-related adverse events

All adverse events occurring in the 48 AGC patients during apatinib combined with ICIs treatment were systematically analyzed. Overall, treatment-related toxicities were observed in 39 out of 48 patients (81.25%), with most toxicities showing similar patterns between groups. As presented in **Table 3**, the monotherapy group commonly experienced fatigue (36%), nausea/vomiting (24%), hypoproteinemia (20%), hand-foot syndrome (HFS, 16%), albuminuria (16%), rash (12%), and elevated transaminases (12%). The apatinib-ICIs combination group frequently reported albuminuria (24%), fatigue (20%), elevated transaminases (16%), hyperbilirubinemia (16%), diarrhea (16%), and granulocytopenia (12%). The combined regimen demonstrated acceptable tolerability, with 23 patients maintaining manageable toxicity profiles.

**Table 3:** The adverse events between two groups.

Items	Apatinib N=25			Apatinib + ICIs N=23		
Treatment-related toxicities	I-II	≥III	Sum	I-II	≥III	Sum
Palpitation	0	0	0	1 (4%)	0	1 (4%)
Hypertension	0	0	0	1 (4%)	1 (4%)	2 (8%)
Leukopenia	2 (8%)	0	2 (8%)	2 (8%)	0	2 (8%)
Anaemia	0	1 (4%)	1 (4%)	1 (4%)	1 (4%)	2 (8%)
Granulocytopenia	2 (8%)	0	2 (8%)	2 (8%)	1 (4%)	3 (12%)
Fatigue	8 (32%)	1(4%)	9 (36%)	5 (20%)	0	5 (20%)
Elevated ransaminases	2 (8%)	1(4%)	3 (12%)	4 (16%)	0	4 (16%)
Hyperbilirubinemia	1(4%)	1(4%)	2 (8%)	2 (8%)	2 (8%)	4 (16%)
Hypoproteinemia	5 (20%)	0	5 (20%)	1 (4%)	0	1 (4%)
Nausea/vomiting	6 (24%)	0	6 (24%)	1 (4%)	0	1 (4%)
Celialgia	2 (8%)	0	2 (8%)	1 (4%)	0	1 (4%)
Diarrhea	3 (24%)	0	3 (12%)	4 (16%)	0	4 (16%)
Gastrointestinal hemorrhage	1(4%)	0	1 (4%)	0	1 (4%)	1 (4%)
Increased creatinine	0	0	0	0	0	0
Albuminuria	4 (16%)	0	4 (16%)	6 (24%)	0	6 (24%)
Rash	2 (8%)	1 (4%)	3 (12%)	2 (8%)	0	2 (8%)
Hand-foot syndrome	3 (12%)	1 (4%)	4 (16%)	1 (4%)	0	1 (4%)
Dysglycemia	0	1 (4%)	1 (4%)	0	0	0

### Adjustment by using univariate analysis and multivariable cox regression analysis

We primarily examined the influence of several factors on treatment prognosis, including age (≤45 years, 45–60 years, ≥60 years), gender (male/female), primary tumor site (cardia/body of stomach/other), ECOG score (0–1/≥2), liver metastasis (yes/no), peritoneal metastasis (yes/no), co-occurrence of signet-ring cell carcinoma (SRCC) (yes/no), oligometastasis (yes/no), response evaluation (progressive disease [PD]/partial response [PR]/stable disease [SD]), initial dosage of apatinib (250 mg/500 mg), and use of combined ICIs (yes/no). (**Table 4**) Univariate analysis identified five significant factors: better ECOG status (0-1; HR=0.4, *P*=0.003), liver metastases presence (HR=0.52, *P* =0.042), peritoneal metastases presence (HR=2.66, *P* =0.002), SD response (HR=0.45, *P* =0.022), and ICI combination (HR=0.44, *P* =0.009). Multivariate analysis confirmed ECOG≥2 (HR=0.3, p=0.0009) and peritoneal metastases (HR=2.6, *P* =0.0096) as independent prognostic factors.

**Table 4:** Univariate analysis and multivariable cox regression analysis.

	Univariate analysis			Multivariate analyses		
Factors	HR	HR 95%CI	*P*	HR	95%CI	*P*
Age						
≤45	—					
45-60	1.4	0.49-3.8	0.549			
≥60	1.3	0.49-3.4	0.619			
Gender						
F	—					
M	1.26	0.69-2.32	0.448			
Primary Tumor Site						
Cardia	—					
Body of Stomach	1.7	0.69-4.19	0.246			
Others	1.01	0.50-2.05	0.978			
ECOG Score						
≥2	—					
0-1	0.4	0.22-0.74	0.003	0.3	0.15-0.61	0.0009
Liver metastasis						
N	—					
Y	0.52	0.28-0.98	0.042	0.49	0.23-1.04	0.063
Peritoneal metastasis						
N	—					
**Y**	2.66	1.43-4.94	0.002	2.6	1.26-5.35	0.0096
Combined SRCC						
N	—					
Y	1.29	0.59-2.82	0.524			
Oligometastasis						
N	—					
Y	1.31	0.72-2.4	0.379			
Response evaluation						
PD	—					
PR	0.25	0.03-1.96	0.187	0.91	0.1-8.15	0.935
SD	0.45	0.23-0.89	0.022	0.57	0.27-1.20	0.137
Initial dosage (Apatinib)						
250mg						
500mg	1	1	0.437			
Combined ICIs						
N	—					
Y	0.44	0.24-0.82	0.009	0.61	0.3-1.22	0.16

“Others” included: gastric antrum, gastroesophageal junction/fundus, diffuse-type.

### Comparison of PFS and OS in subgroups

**Figure 3:**
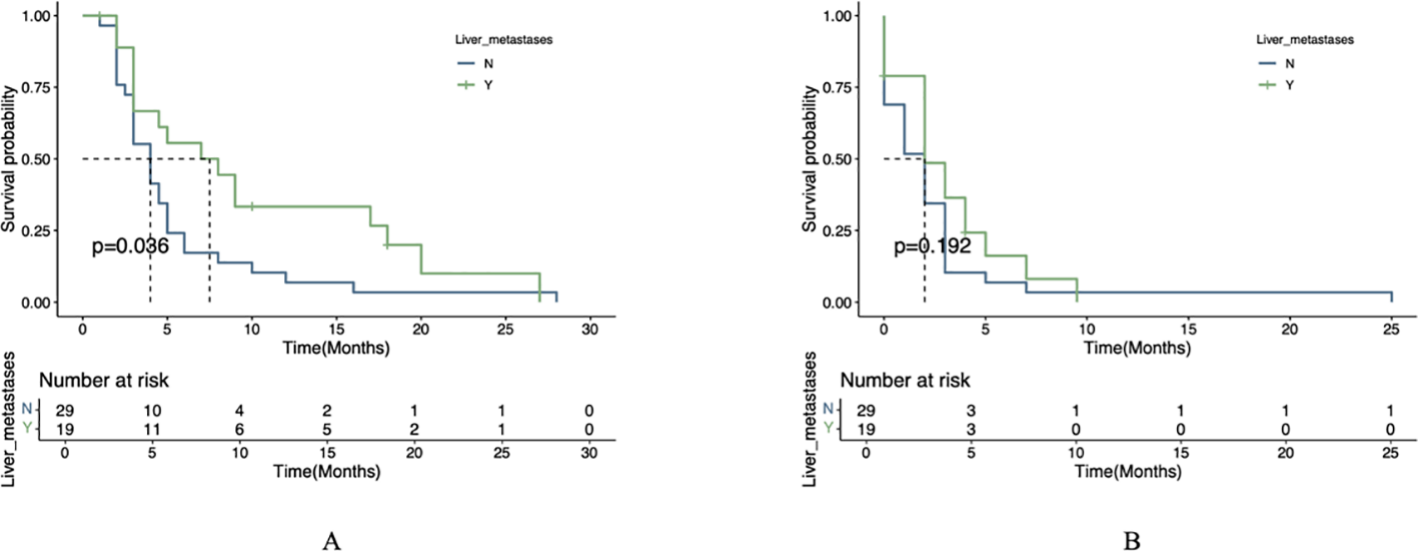
Survival analysis stratified by liver metastasis status. **(A)** OS comparison between patients with versus without liver metastases. **(B)** PFS comparison between patients with versus without liver metastases.

In the exploratory subgroup analysis, patients with liver metastases receiving combination therapy showed significantly longer median OS (7.5 vs. 4.0 months, *P*=0.036), though no significant PFS benefit was observed (*P*=0.192) (**Figure 3**). This trend aligns with preclinical studies suggesting enhanced drug delivery to liver metastases due to their arterial-dominant blood supply, but further validation is required. Similar trends were reported in other trials, such as the AVAGAST study (HR=0.63 for PFS in liver metastases) (12) and REGONIVO trial (mPFS=5.6 months with anti-angiogenic/ICIs combination), though none directly confirmed hemodynamic mechanisms.

**Figure 4:**
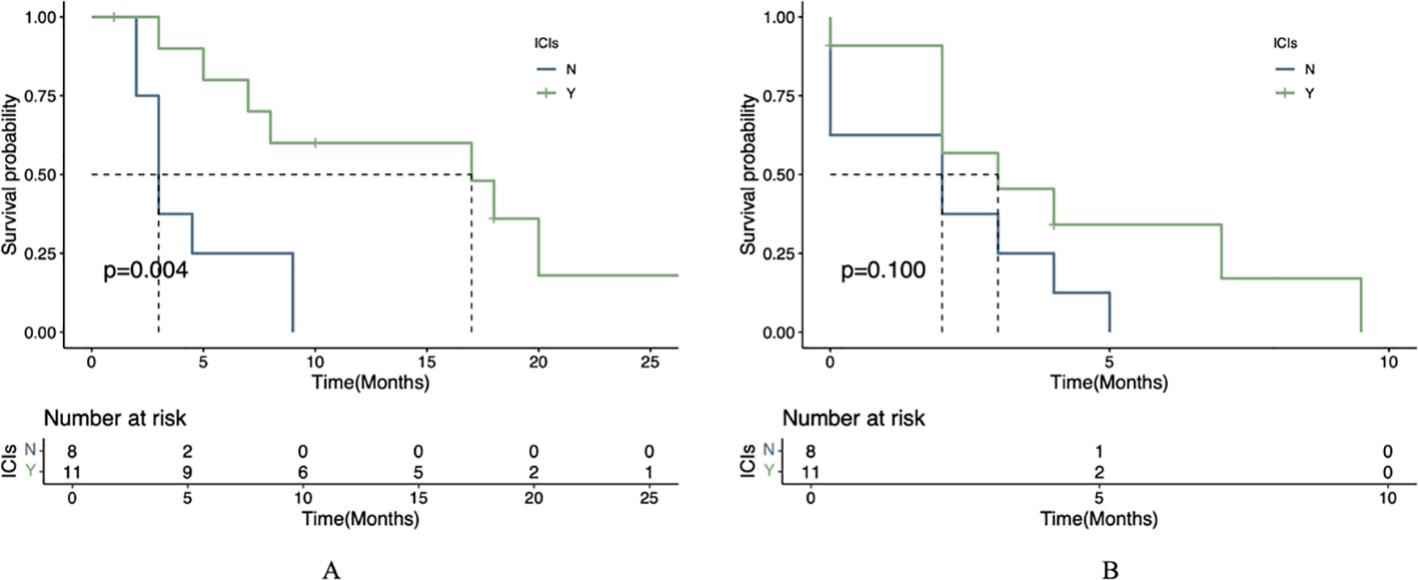
Therapeutic efficacy in liver metastasis cohort. **(A)** OS in patients receiving apatinib plus immunotherapy versus apatinib alone. **(B)** PFS in patients receiving apatinib plus immunotherapy versus apatinib alone.

Statistical analysis using the log-rank test revealed significant differences in survival outcomes between treatment groups for patients with liver metastases. The combination therapy group (apatinib plus ICIs) demonstrated significantly longer mOS compared to the apatinib monotherapy group (*P*=0.004). However, no statistically significant difference was observed in mPFS between the two groups (*P*=0.1; **Figure 4**).

## A Case report:a case of long-term survival in agc utilizing immunotherapy combined with apatinib

A 52-year-old male patient was diagnosed with GC in July 2018 and underwent radical gastrectomy the same month. The postoperative pathology revealed poorly differentiated adenocarcinoma of the stomach, measuring approximately 6cm by 4.5cm, infiltrating the outer fibroadipose layer. Lymph node dissection results were as follows: 0/8 at the cardia, 5/14 at the lesser curvature, 0/2 at the greater curvature, and 0/4 at the subpylorus, with metastasis detected in the 11th group of lymph nodes. No tumor invasion was observed in the greater omentum. The patient was staged postoperatively as T4aN2M0, stage IIIB, and initially received no chemotherapy.

**Figure 5:**
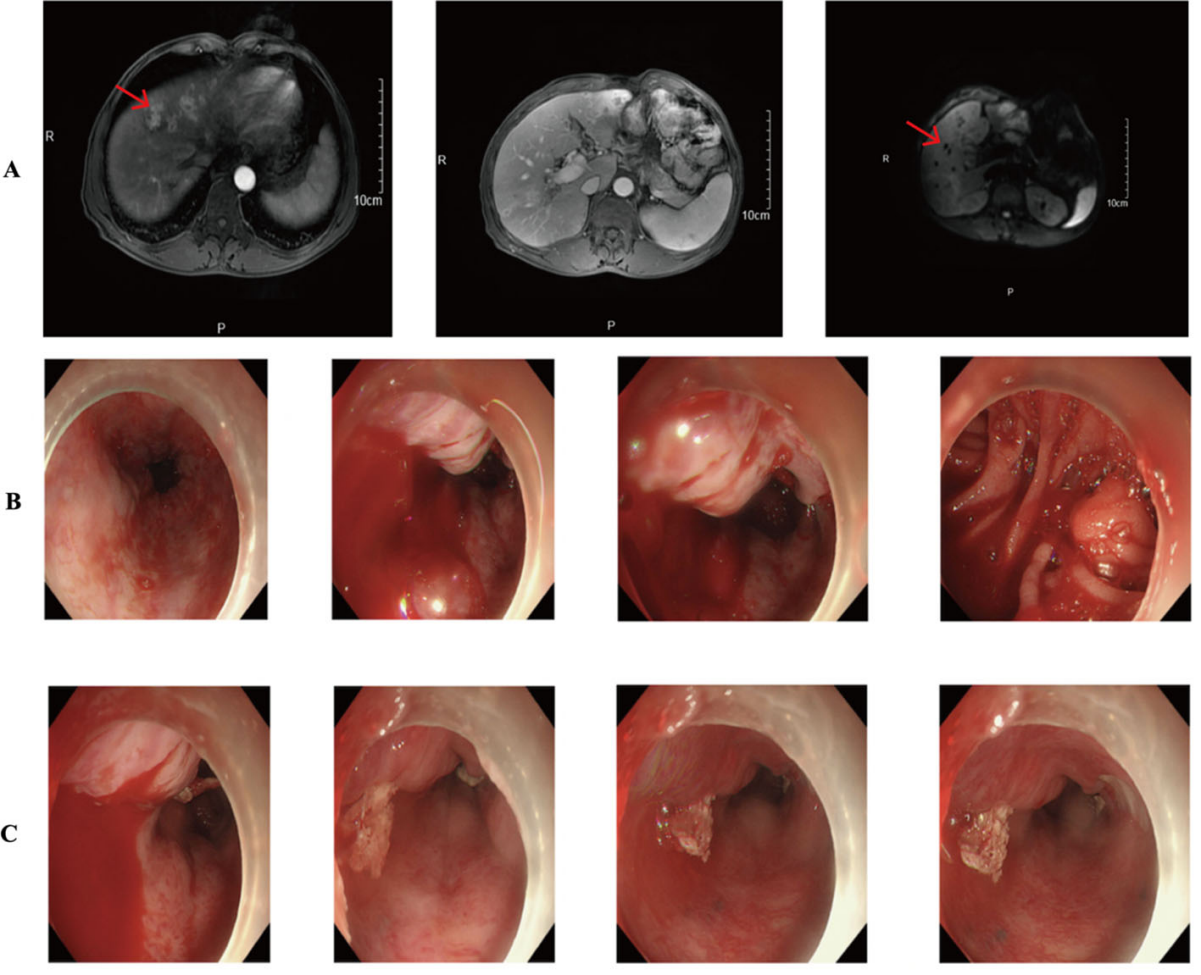
**(A)** CT results showed multiple intrahepatic metastases. **(B, C)** Gastroscopy suggests active gastrointestinal bleeding.

In March 2019, the patient developed abdominal discomfort and elevated tumor markers (CEA: 100μg/L). CT imaging indicated retroperitoneal recurrence, prompting the initiation of three cycles of SOX chemotherapy from March to May 2019. Subsequent evaluations showed no significant decrease in tumor markers, and a liver metastasis was identified (**Figure 5A**). The treatment regimen was then changed to three cycles of albumin-bound paclitaxel combined with S-1 from May to July 2019, which failed to control the disease effectively.

On September 9, 2019, the patient commenced a combined treatment regimen of Carrilizumab, apatinib, and albumin-bound paclitaxel. After one cycle, impaired liver function led to his transfer to another hospital, where an MRI revealed multiple liver nodules and retroperitoneal lymph node metastases. A liver biopsy confirmed the metastases with immunohistochemistry results showing EBER(-), Her-2(+), MSH6(+), MSH2(+), MLH1(+), and PMS2(+).

**Figure 6:**
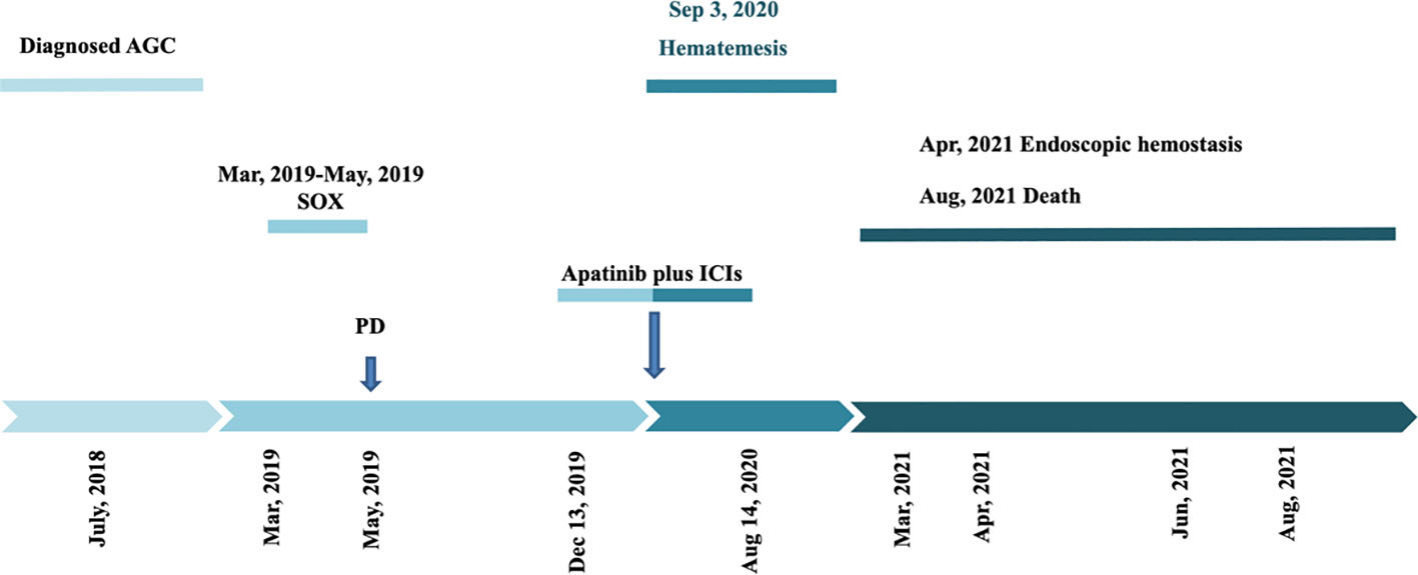
Patient survival timeline as illustrated.

The patient was subsequently admitted to our hospital and completed ten cycles of PD-1 inhibitor therapy combined with apatinib from December 13, 2019, to August 14, 2020. In September 2020, he presented with hematemesis, which was managed with endoscopic hemostasis, intravenous proton pump inhibitor therapy, and nil per os (NPO) status, resulting in clinical improvement (**Figures 5B, C**). Apatinib was discontinued on October 20, 2020, while PD-1 inhibitor treatment was maintained for one additional cycle. The patient required multiple hospitalizations in the gastroenterology department for endoscopic management of esophageal varices in January, April, and June 2021. The patient expired in October 2021 (**Figure 6**), with an OS of 20 months and PFS of 9 months.

## Discussion

In recent years, there has been growing interest in exploring the potential synergistic effects between ICIs and tyrosine kinase inhibitors (TKIs) to enhance tumor immunotherapy outcomes (13–16). Anti-angiogenic therapy promotes vascular normalization in tumors, alleviating hypoxia, improving drug delivery, and remodeling the TME to enhance immunotherapy efficacy (17). PD-1/PD-L1 inhibitors reinstate an immune-permissive microenvironment by activating effector T cells and augmenting IFN-γ production, which in turn promotes tumor vascular normalization and potentiates T cell infiltration and cytotoxic activity (18). Collectively, these therapies exhibit synergistic effects in promoting tumor vascular normalization and remodeling the TME, while simultaneously reducing the requisite immunotherapy dosage and mitigating treatment-related adverse events (19). In the pivotal phase III CARES-310 trial investigating advanced hepatocellular carcinoma, patients in the experimental arm received combination therapy with carrelizumab (200 mg administered intravenously every two weeks) plus apatinib (250 mg orally once daily), while the control arm received sorafenib (400 mg orally twice daily) as the standard molecular-targeted therapy. The study demonstrated significant clinical benefits, with the experimental group achieving a mPFS of 5.6 months versus 3.7 months in the control group (hazard ratio [HR] = 0.52; 95% CI: 0.41-0.65; *P* < 0.0001), representing a 48% reduction in the risk of disease progression or death. Furthermore, the combination therapy showed superior OS outcomes, with a mOS of 22.1 months compared to 15.2 months in the control group (HR = 0.62; 95% CI: 0.49-0.80; *P* < 0.0001), corresponding to a 38% reduction in mortality risk (20). In a Phase II clinical trial investigating pembrolizumab combined with the anti-angiogenic agent lenvatinib in AGC, the combination therapy demonstrated remarkable efficacy. The ORR reached 69% when used as either first-line or second-line treatment. Notably, the DCR achieved 100%. The regimen showed durable clinical benefit with a mPFS of 7.1 months and an impressive 12-month OS rate exceeding 70% (21). In a Phase Ib clinical trial evaluating the safety and efficacy of regorafenib-nivolumab combination therapy in advanced gastric or colorectal cancer patients, the cohort with gastric cancer demonstrated a mPFS of 5.6 months and a mOS of 12.3 months (22).

In this study, we compared the efficacy and tolerability of apatinib combined with ICIs versus apatinib monotherapy. The observed dissociation between significant OS benefit (6.0 vs. 3.0 months, *P*=0.009) and non-significant PFS improvement (3.0 vs. 2.0 months, *P*=0.155) with combination therapy may be attributed to multiple factors. First, the characteristic ‘tail effect’ of ICIs - where immune-mediated tumor control develops gradually - could drive OS superiority despite modest PFS gains, as demonstrated in other immunotherapy trials (23). Second, differential post-progression treatment patterns, including more frequent use of subsequent immunotherapy in the combination group, may have further extended survival independently of initial PFS outcomes (24). Finally, our study may have been underpowered to detect the clinically relevant 1-month PFS difference (HR=0.44) given the small cohort size (n=48) and limited event numbers, though the trend remains biologically plausible given apatinib ‘s known vascular normalization effects. In the univariate prognostic analysis, the results demonstrated that ICIs, liver metastasis, and peritoneal metastasis were significant prognostic factors influencing OS. Subsequently, we performed a multivariate Cox regression analysis; however, the combination of apatinib with ICIs did not emerge as an independent prognostic factor for OS. This observation may be attributed to the limitations of our study, which was a single-center investigation with a relatively small sample size. Surprisingly, the presence of liver metastasis was associated with improved OS, a finding that warrants further investigation. Preclinical studies suggest that arterial-dominant blood flow in liver metastases could enhance delivery of anti-angiogenic agents like apatinib (25), while synergistically remodeling the immunosuppressive TME (9). However, clinical data directly linking this anatomic feature to drug distribution are lacking in gastric cancer. While our study highlights the survival benefits of apatinib -ICI combination therapy, particularly in patients with liver metastases, it is important to recognize that not all AGC patients respond equally to this regimen. Emerging evidence suggests that genomic features such as microsatellite instability-high (MSI-H) status, PD-L1 expression (CPS ≥1 or ≥5), and HER-2 amplification may predict better responses to immunotherapy (3, 4, 23, 26). Additionally, tumor mutational burden (TMB) and specific alterations in pathways like the VEGF/VEGFR axis or immune-related genes could further stratify patients likely to benefit from combination therapy (27–29). Future studies should incorporate comprehensive molecular profiling to validate these biomarkers and optimize patient selection. However, as a single-center real-world study with a limited sample size, further large-scale research is required to validate these findings. We performed a subgroup analysis in gastric cancer patients with liver metastasis, which demonstrated that apatinib combined with ICIs yielded a more favorable prognosis compared to apatinib monotherapy. Regarding safety, most treatment-related adverse events (TRAEs) were mild and manageable. No significant differences in the incidence of TRAEs (any grade) were observed between the apatinib plus PD-1 mAb group and the apatinib monotherapy group. The most common adverse events included fatigue, proteinuria, and transaminase elevation, all of which were generally manageable. Furthermore, we report a representative case in which combination therapy with apatinib and PD-1 antibody achieved a PFS of 9 months, demonstrating the potential efficacy of this regimen in prolonging patient survival. This case also exemplifies the gastrointestinal bleeding risk associated with anti-angiogenic therapy. Future studies should emphasize personalized treatment strategies and investigate gastrointestinal bleeding risks, particularly in gastric cancer patients who develop portal hypertension or other specific complications following liver metastasis.

## Study limitations

This study has several limitations. First, the small sample size (n=48) from a single institution restricts statistical power and generalizability, particularly for secondary endpoints such as PFS. Although the combination therapy demonstrated promising efficacy—especially in liver metastasis patients—these results require validation in larger multicenter trials. Second, while preclinical data and prior trials (e.g., AVAGAST) indirectly support hepatic hemodynamics’ role in drug delivery, direct clinical evidence in gastric cancer remains scarce. Further prospective studies incorporating pharmacokinetic imaging are warranted to validate both therapeutic benefits and mechanistic hypotheses. Third, despite multivariable adjustments, baseline ECOG performance status imbalance (74% vs. 40% ECOG 0–1 in combination vs. monotherapy groups) may have confounded survival outcomes, as clinicians preferentially assigned fitter patients to combination therapy. Future studies should employ propensity-matched cohorts or interaction analyses to better control for performance status heterogeneity.

## Conclusion

Our study demonstrates that ICIs combined with apatinib provides superior survival benefits over apatinib monotherapy in AGC patients, with a manageable safety profile. The enhanced efficacy observed in liver metastasis cases may relate to the liver’s abundant vascular supply, unique hemodynamic characteristics, and specialized tumor microenvironment. While bleeding risk warrants vigilance, this combination regimen represents a promising third-line option for AGC, pending further large-scale validation.

## Data Availability

The original contributions presented in the study are included in the article/supplementary material, further inquiries can be directed to the corresponding authors.
